# Excitability decreasing central motor plasticity is retained in multiple sclerosis patients

**DOI:** 10.1186/1471-2377-12-92

**Published:** 2012-09-13

**Authors:** Daniel Zeller, Su-Yin Dang, David Weise, Peter Rieckmann, Klaus V Toyka, Joseph Classen

**Affiliations:** 1 Dept. of Neurology, University of Würzburg, Josef-Schneider-Str. 11, 97080, Würzburg, Germany; 2 Dept. of Neurology, University of Leipzig, 04103, Leipzig, Germany; 3 Neurologische Klinik Bamberg, 96049, Bamberg, Germany

**Keywords:** Multiple sclerosis, LTD, Motor plasticity, TMS, Motor cortex

## Abstract

**Background:**

Compensation of brain injury in multiple sclerosis (MS) may in part work through mechanisms involving neuronal plasticity on local and interregional scales. Mechanisms limiting excessive neuronal activity may have special significance for retention and (re-)acquisition of lost motor skills in brain injury. However, previous neurophysiological studies of plasticity in MS have investigated only excitability enhancing plasticity and results from neuroimaging are ambiguous. Thus, the aim of this study was to probe long-term depression-like central motor plasticity utilizing continuous theta-burst stimulation (cTBS), a non-invasive brain stimulation protocol. Because cTBS also may trigger behavioral effects through local interference with neuronal circuits, this approach also permitted investigating the functional role of the primary motor cortex (M1) in force control in patients with MS.

**Methods:**

We used cTBS and force recordings to examine long-term depression-like central motor plasticity and behavioral consequences of a M1 lesion in 14 patients with stable mild-to-moderate MS (median EDSS 1.5, range 0 to 3.5) and 14 age-matched healthy controls. cTBS consisted of bursts (50 Hz) of three subthreshold biphasic magnetic stimuli repeated at 5 Hz for 40 s over the hand area of the left M1. Corticospinal excitability was probed via motor-evoked potentials (MEP) in the abductor pollicis brevis muscle over M1 before and after cTBS. Force production performance was assessed in an isometric right thumb abduction task by recording the number of hits into a predefined force window.

**Results:**

cTBS reduced MEP amplitudes in the contralateral abductor pollicis brevis muscle to a comparable extent in control subjects (69 ± 22% of baseline amplitude, p < 0.001) and in MS patients (69 ± 18%, p < 0.001). In contrast, post-cTBS force production performance was only impaired in controls (2.2 ± 2.8, p = 0.011), but not in MS patients (2.0 ± 4.4, p = 0.108). The decline in force production performance following cTBS correlated with corticomuscular latencies (CML) in MS patients, but did not correlate with MEP amplitude reduction in patients or controls.

**Conclusions:**

Long-term depression-like plasticity remains largely intact in mild-to-moderate MS. Increasing brain injury may render the neuronal networks less responsive toward lesion-induction by cTBS.

## Background

Several distinct mechanisms are believed to contribute to the compensation of brain injury in multiple sclerosis (MS). Apart from processes of tissue repair at the cellular level, neuronal plasticity may play a major role [1-6]. Synapse-specific Hebbian forms of plasticity, such as long-term potentiation (LTP) and depression (LTD), are supposed to represent potentially rate-limiting steps on the way to successful long-term reorganization of the brain [7,8]. Rapid onset plasticity of neuronal connections likely involves both enhancement and depression of synaptic efficacy. We previously showed that LTP-like motor plasticity remains intact in mild-to-moderately afflicted MS patients [9]. However, it remains unknown if excitability decreasing, i.e. LTD-like plasticity is compromised in MS patients. This type of plasticity may be particularly important because it is challenged when neuronal activity needs to be focussed during the process of acquiring or regaining a specific skill. Functional magnetic resonance (fMRI) studies addressing short-term adaptation, i. e. the attenuation of the fMRI response during repeated motor task execution, provided inconsistent results. When studying fMRI activation patterns that were associated with performance of voluntary thumb movements before and after motor training, Morgen and colleagues [5] found that MS patients lack the typical task-specific training-dependent reductions in activation of some contralateral cortical regions that they observed in healthy controls. However, in another recent fMRI study, regional brain activation induced by externally cued right hand tapping decayed normally over consecutive runs in MS patients [10]. The extent of this decay was not influenced by a modest disease progression observed over one year [10]. Thus, an assessment of LTD-like plasticity in MS by neurophysiological means is of special interest because it taps more directly into mechanisms limiting neuronal excitation and might potentially translate into behavioural treatment strategies.

In the present study, we tested LTD-like rapid-onset central motor plasticity in patients with MS and in healthy controls using an excitability-decreasing transcranial magnetic stimulation protocol (continuous theta-burst stimulation, cTBS). cTBS has previously been shown to induce a depression of corticospinal excitability whose physiological properties resemble those observed for LTD as studied in animal preparations [11-13]. The effects of the cTBS intervention were assessed neurophysiologically (motor evoked potentials, MEPs) and behaviourally (force production performance, FPP). We examined whether LTD-like rapid-onset central motor plasticity and responsiveness toward an interfering intervention over the primary motor cortex (M1) are compromised in patients with MS.

## Methods

### Patients and healthy controls

Fourteen patients with definite MS aged between 23 and 48 years (30.1 ± 6.8 years, mean ± SD) were recruited from the outpatient clinic of the Clinical Research Group for MS at the Department of Neurology, University of Würzburg. Eleven of these patients had already been included in a previous study [14]. The present experiments were done at least six weeks after those previous tests. The recruiting neurologist was not informed about the scientific hypothesis of this study as to avoid any selection bias. A full neurologic examination was done including the Expanded Disability Status Scale (EDSS) [15]. Patients were eligible for the study if the following inclusion criteria were met: 1) age between 18 and 60 years, 2) stable clinical condition within the past three months (i. e. absence of relapse, progression, or changes in therapy), 3) no medication targeting α-adrenergic or serotonergic receptors, and 4) exclusion of pregnancy by lab testing. All MS patients were right-handed, according to a modified version of the Edinburgh Inventory. Fourteen healthy controls recruited from a large database of volunteers were matched for age, sex, and handedness. Clinical characteristics of MS patients and controls are summarized in Table 1. 

**Table 1:** Clinical characteristics of MS patients and controls

**Patient no.**	Age* (years)	Sex*	**Duration of MS (years)**	**Clinical subtype**	**Current DMT**	**EDSS**	**Control no.**	**Age (years)**	**Sex**
1	23	m	4	RRMS	(FTY/Plac)	2.0	1	26	m
2	23	m	7	RRMS	FING	2.0	2	27	m
3	32	m	9	RRMS	IF	1,0	3	27	m
4	32	m	6	RRMS	GA	0.0	4	33	m
5	34	m	4	RRMS	NAT	3.5	5	35	m
6	48	m	3	RRMS	IF	2.0	6	45	m
7	23	f	6	RRMS	NAT	1.5	7	24	f
8	25	f	6	RRMS	IF	1.5	8	26	f
9	25	f	8	RRMS	IF	1.5	9	26	f
10	28	f	7	RRMS	IF	2.0	10	26	f
11	29	f	1	RRMS	IF	1.0	11	26	f
12	31	f	3	RRMS	IF	1.0	12	28	f
13	33	f	9	RRMS	IF	2.5	13	33	f
14	36	f	9	RRMS	IF	1.5	14	45	f
Mean	30.1		5.9		Median	1.5	Mean ±	30.5	
± SD	± 6.8		± 2.5		[range]	[0–3.5]	SD	± 6.9	

*Patients listed by gender and age.

m = male; f = female; EDSS = expanded disability status scale; RRMS = Relapsing-Remitting MS; SPMS = Secondary Progressive MS; DMT = disease modifying therapy with immunomodulators; FING = fingolimod; GA = glatiramer acetate; IF = interferon beta; MIT = mitoxantrone; NAT = natalizumab; (FTY/Plac) = fingolimod or placebo; patient participated in a randomized controlled treatment trial with undisclosed allocation.

The study conformed to the principles of the declaration of Helsinki. It was approved by the Ethics committee of the Medical Faculty at the University of Würzburg. All MS patients and control subjects gave their written informed consent for this research study.

### Transcranial magnetic stimulation (TMS) and EMG recording

#### *Stimulation*

Focal TMS was performed using a figure-of-eight shaped magnetic coil (C-B60 Medtronic) connected to a MagPro X100 magnetic stimulator (Medtronic A/S 2740 Skovlunde, Denmark). The pulse shape was either monophasic or biphasic, as indicated below. The coil was held tangentially to the skull with the handle pointing backwards and laterally at a 45° angle to the sagittal plane. The optimal position of the magnetic coil for eliciting MEPs in the abductor pollicis brevis (APB) muscle of the dominant hand was assessed over the left M1 and digitally recorded with a neuronavigational device (see below). At this position, termed “motor hot-spot”, the resting motor threshold (RMT) was determined [16]. Complete relaxation of the ABP was continuously monitored by visual and auditory feedback from the surface EMG. A neuronavigational device (Brainsight, Rogue Research, Montreal, Canada) was used to increase the fidelity of stably positioning the TMS coil over the course of an experiment.

#### *Electromyographic recordings*

Surface EMG activity was recorded from the right APB muscle using surface electrodes in a belly-tendon montage. Raw signals were amplified using a differential amplifier (CED 1902, Cambridge Electronic Design, Cambridge, UK) and bandpass-filtered between 1 and 2000 Hz. EMG signals were sampled at 5000 Hz, digitized using an analogue–digital converter (CED 1401 plus, Cambridge Electronic Design, Cambridge, UK) and stored in a laboratory computer.

### Continuous theta-burst stimulation (cTBS)

cTBS was performed similar to the protocol described by Huang et al. [12]. Trains of magnetic pulses containing 3 TMS pulses of 50 Hz (i. e. with an interval of 20 ms between each stimulus) were repeated at 200 ms intervals (i. e., 5 Hz) for a duration of 40 sec (total of 600 pulses). Stimulus intensity was set to 0.7 x RMT as assessed in the right APB muscle [17]. In control subjects, an additional “sham” stimulation at 2% of the maximal stimulator output was performed in a pseudorandomized and counterbalanced design. For cTBS, the pulse shape was biphasic. As a safety measure, the EMG of biceps brachii muscle was continuously monitored during cTBS to detect potential spread of excitation to proximal muscles.

### Force production performance (FPP)

**Figure 1:**
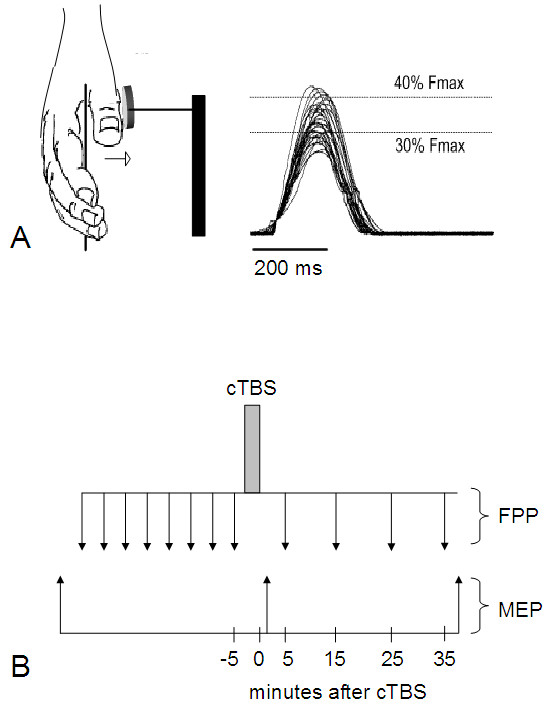
** (A) Representative force trajectories (on right) obtained with isometric right thumb abductions (abductor pollicis brevis muscle, ABP; on left).** Force production performance was assessed by the number of attempts falling within the target force window displayed as two horizontal lines on the computer screen. Fmax: individual maximum force. (**B**) Schematic overview of the experimental procedure. cTBS, continuous theta-burst stimulation; MEP, motor-evoked potentials; FPP, force production performance.

FPP was assessed as described previously [18]. Briefly, subjects performed brisk isometric abductions with the right thumb against a force transducer (Grass CP122A, Grass Instruments CO, West Warwick, RI). The force transducer was adjusted such that it could be easily and immediately activated by pure thumb abduction movements. First, the subject’s maximum force was obtained as the mean of five consecutive trials of maximal force production. A target force window was then defined as the range between 30% and 40% of the subject’s maximum force. The subject performing the test was asked to focus on the computer screen and instructed to position the force curve between the two horizontal lines by appropriate abductor muscle contraction (Figure 1A). Each block consisted of 30 metronome paced isometric thumb abductions at 0.5 Hz. The number of successful attempts falling within the target force window was taken as a measure of performance. 

### Experimental procedure

Patients and control subjects were seated comfortably in an armchair. The cortical “motor hot-spot” was obtained as described above. The functionally defined hand motor cortex position, which corresponded very closely with the anatomical location according to the landmarks described previously [19], was used to mark the final M1 stimulation position in the digital data set. At this site, the RMT was determined for both monophasic and biphasic pulse shape.

Corticospinal excitability of the APB muscle representation was probed by collecting 30 TMS pulses at a stimulus intensity of 1.3 x RMT and a stimulation rate of 0.1 Hz. The pulse shape was monophasic. MEP responses were recorded from the right APB muscle. To optimize FPP before any intervention, each subject performed eight training blocks, separated by one minute to avoid fatigue. Thereafter, cTBS was applied to the left M1 area. Thirty MEP responses were collected immediately (t1) and at 40 min (t2) after cTBS. FPP was established at 5 min, 15 min, 25 min, and 35 min after cTBS (Figure 1B).

### Data analysis

*FPP* was assessed by the number of successful attempts (within a block of 30 trials) falling within the target force window. In order to assess the net effect of cTBS on FPP, to minimize the influence of unspecific factors such as differences in baseline performance between subjects, and to account for learning effects and incremental performance over the course of the session, we used the FPP values of the mean of each of the eight blocks acquired before cTBS intervention to compute a performance trendline. Postinterventional performance was compared against the extrapolation of this trendline. cTBS-induced changes were estimated as deviations from the linear trend of pre-interventional performance (“delta”). For comparison with the trendline, performance was tested against unity using two-tailed one-sample t-tests. The false discovery rate (FDR) correction was applied to control for multiple comparisons.

*MEP amplitudes* were measured peak-to-peak in each individual trial. Each block of 30 MEP amplitudes was averaged. To assess the effect of cTBS intervention on cortical excitability, changes of MEP amplitude were expressed as percent difference from baseline. Repeated measure analyses of variance (ANOVA_RM_) were used for statistical analysis, and two-tailed t-tests for post hoc analysis. Effects were considered significant if p < 0.05. If not stated otherwise, all values are given as means ± SD.

## Results

Demographic and clinical features of MS patients are summarized in Table 1. As expected, corticomuscular latency (CML) to the APB muscle of the right hand in MS patients tended to be increased as compared to healthy controls (see Table 2).

**Table 2:** Baseline measurements in MS patients and controls

**Motor test**	**MS patients**	**Controls**	**p value**
Corticomuscular latency, CML (msec)	21.9 ± 1.7	20.8 ± 1.0	0.052
Force production performance at baseline	14.3 ± 3.5	18.4 ± 4.1	0.009
Resting motor threshold, monophasic (%)	54.2 ± 10.9	50.8 ± 11.6	0.428
Resting motor threshold, biphasic (%)	37.4 ± 5.6	41.6 ± 11.7	0.236
MEP amplitudes at baseline (mV)	1.5 ± 1.0	1.2 ± 0.8	0.450

MEP = motor evoked potential.

### Changes of corticospinal excitability by cTBS

#### *Baseline measurements*

RMT and MEP amplitudes in the APB muscle before cTBS were comparable between MS patients and age-matched controls (see Table 2).

#### *cTBS-induced effects*

**Figure 2:**
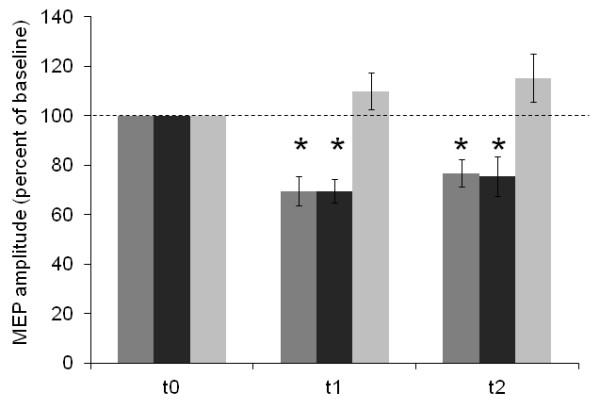
** Changes in baseline-normalized MEP amplitudes of the APB induced by cTBS (%) in 14 MS patients (dark grey columns) and 14 matched control subjects (real cTBS: medium grey columns; sham cTBS: light grey columns).** Error bars indicate the standard error of the mean (SEM). Asterisks indicate significant difference from baseline.

Changes in baseline-normalized MEP amplitudes of APB induced by cTBS are illustrated in Figure 2. In healthy controls, cTBS of the left M1 modulated the magnitude of MEP amplitudes of the contralateral APB muscle depending on the stimulation mode. ANOVA_RM_ (MODE (real, sham) x TIME (t0,t1,t2)) revealed a significant MODE × TIME interaction (F(2,52) = 10.8; p < 0.001) while the (within-subjects) factor TIME was not significantly different. Post hoc testing revealed that there was a significant suppression of contralateral MEP amplitudes when real cTBS (0.7 x RMT) was applied (t1: 69 ± 22%; p < 0.001; t2: 77 ± 21%; p = 0.001) as opposed to sham cTBS (t1: 110 ± 28%; p = 0.203; t2: 115 ± 36%; p = 0.142).

In MS patients and controls, cTBS (0.7 x RMT) of the left M1 induced a decrease of the MEP amplitudes of the contralateral APB. ANOVA_RM_ (HEALTH (multiple sclerosis, MS; controls, CTRL) x TIME (t0,t1,t2)) revealed significant effects of the (within-subjects) factor TIME (F(2,52) = 27.3, p < 0.001), but not of the TIME x HEALTH interaction (F(2,52) = 0.2, p = 0.985). The (between-subjects) factor HEALTH (F(1,26) = 0.01, p = 0.936) was not significant. Upon exploratory post hoc testing, cTBS effects were found significant for epochs t1 (69 ± 22%, p < 0.001) and t2 (77 ± 21%, p = 0.001) in controls, and similarly for epoch t1 (69 ± 18%, p < 0.001) and t2 (75 ± 30, p = 0.008) in MS patients.

### Changes of force production performance (FPP) by cTBS

**Figure 3:**
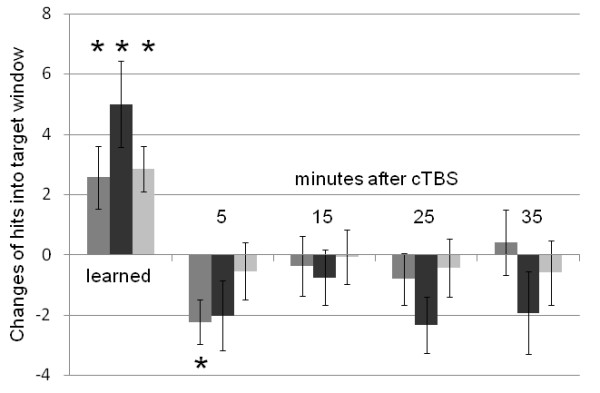
** Changes of force production performance over the course of the eight training blocks (“learned”) and at four time points following cTBS in 14 MS patients (dark grey columns) and 14 matched control subjects (real cTBS: medium grey columns; sham cTBS: light grey columns).** Error bars indicate the standard error of the mean (SEM). Asterisks indicate significant difference from baseline after proper training (see methods; “learned”) and from the extrapolated performance trendline at different time intervals (in minutes; “after cTBS”), respectively (two-tailed, one-sample *t*-test, after FDR correction).

At baseline, MS patients performed worse than controls in the force production task (see Table 2). However, over the course of the eight training blocks, improvements of FPP were statistically comparable between MS patients and healthy controls (p = 0.183; see Figure 3), MS patients and controls reached similar performance levels (MS patients: 19.3 ± 4.7; controls: 20.9 ± 2.1; p = 0.240). Following cTBS, FPP showed a significant temporary deterioration in controls (delta: 2.2 ± 2.8, p = 0.011, significant after FDR correction), but not in MS patients (2.0 ± 4.4, p = 0.108) at t1. There were no significant FPP changes following sham cTBS in the control subjects (0.5 ± 3.5, p = 0.579; see Figure 3).

### Correlation of cTBS-induced FPP decline with CML

**Figure 4:**
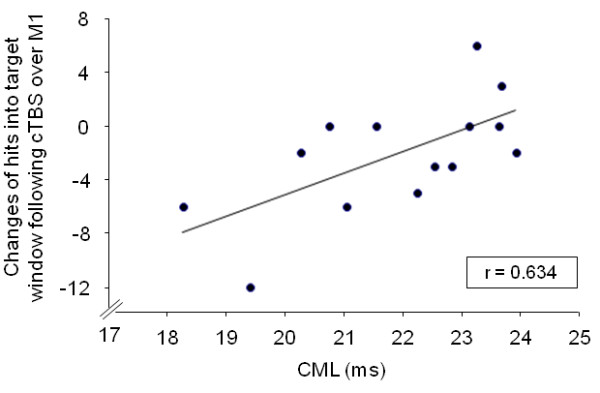
Correlation of changes of the force production performance following cTBS over M1 with corticomuscular latency (CML) in 14 MS patients.

There were no correlations between the changes in baseline-normalized MEP amplitudes and FPP changes following cTBS over M1 in MS patients and controls. However, the FPP decline following cTBS over M1 correlated strongly with CML in MS patients (r = 0.634, p = 0.015; Figure 4), but not in healthy controls.

## Discussion

The present study has examined LTD-like rapid-onset central motor plasticity and functional consequences of a transient virtual M1 lesion induced by cTBS in mild-to-moderately afflicted MS patients and healthy controls. cTBS decreased cortical excitability for a duration of about 30 min in patients and controls to a similar degree. In contrast, cTBS impaired force production performance (FPP) significantly for at least 5 min only in healthy controls, but not in MS patients.

TBS-induced plasticity shares properties with synaptic LTD [11-13] although this has been questioned by some authors [20]. The present findings suggest that LTD-like plasticity remains largely intact in MS patients. Along with previous observations [9], these findings provide evidence that rapid-onset synaptic plasticity as assessed by neurophysiological means is not compromised in mild-to-moderately afflicted MS patients. Previous functional MRI (fMRI) studies probing rapid-onset plasticity in comparably afflicted MS patients provided inconsistent results: One recent fMRI study has demonstrated a decay of regional brain activation by a tapping task over consecutive runs, with similar changes in MS patients and matched controls [10]. In contrast, another study has shown absent task-specific reductions in fMRI activation of some contralateral cortical regions after motor training in MS patients [5]. However, while the first study compared activation patterns of a simple hand tapping paradigm before and after training of this movement, the latter assessed changes in activation for a trained (thumb flexion) relative to an untrained task (thumb extension). Attenuation of the blood-oxygen-level dependent (BOLD) response during repeated execution of an over-learnt motor task may reflect neuronal plasticity, but may also simply result from reduction of attention with time. Therefore, physiological mechanisms cannot be directly inferred from functional imagining studies. By employing a brain stimulation technique, the present study allows a less ambiguous, more direct insight into LTD-like phenomena in MS.

MS-related demyelination, axonal damage, and loss of neuronal synapses may critically interfere with basic prerequisites for the expression of Hebbian as well as cTBS-induced plasticity (see also [9]). In animal models, the endocannabinoid system has been shown to mediate LTD in the neocortex, with CB1 receptors being prominently involved [21,22]. Importantly, the endocannabinoid system is strongly dysregulated in acutely relapsing MS [23]. Therefore, preserved capacity to express LTD-like central motor plasticity may either point to preserved endocannabinoid signalling during stable phases of the disease or to recruitable alternative molecular pathways in MS patients, probably converging towards negative feedback mechanisms at GABAergic and dopaminergic synapses [24].

Although the FPP changes following cTBS over M1 were not statistically significant in MS patients, FPP changes were strongly correlated with CML in this group: Surprisingly, and perhaps counterintuitively, the more CML was prolonged, the less evident was the impairment of FPP by cTBS applied to M1. Although functional activation of non-canonical motor regions may contribute to maintaining motor performance in MS patients [14], it appears highly unlikely that the functional role of M1 has completely be substituted by non-canonical motor regions. Similarly, although the functional redundancy of the motor system (“degeneracy”) might decrease its susceptibility to disruption of one of its nodes (cf. refs. [25,26]), it is unlikely that degeneracy increases with higher lesion load because MS-related pathology does not affect only one node, but multiple brain regions in parallel. We favour, therefore, the alternative possibility that increasing brain injury may render the neuronal networks less responsive toward lesion-induction by cTBS. According to this idea, for TMS to induce behavioural interference, it must act on susceptible brain regions. In healthy people, the temporary behavioral deterioration following off-line cTBS possibly depends on the capacity of cortical circuits to express “noisy oscillations” in response to TMS, thereby decreasing the signal-to-noise ratio of highly organized cortical circuits [27]. In this way, the presence of significant CNS injury (as indexed by prolonged CML) has reduced the capacity of inhibitory interneurons to initiate “noisy oscillations”. This mechanism is consistent with the lack of correlation between cTBS-induced changes of MEP amplitudes and FPP, and provides additional support for conclusions derived from parallel studies of physiology and motor behaviour in healthy subjects [28], showing that mechanisms underlying behavioural effects of cTBS are distinct from those induced at excitatory synaptic connections.

A potential limitation of this study might be the linear extrapolation of the performance trendline, which was used in order to assess the net effect of cTBS on the FPP. However, prolonged motor training was unlikely to lead to stabile performance due to fatigue and/or lapse of concentration in healthy subjects, and possible even much more in MS patients. Decreasing task difficulty was not an alternative option, as this might have resulted in reduced sensitivity of the task towards disruption by TMS. Therefore, our paradigm can be viewed as a trade-off between feasibility, reduced resilience of MS patients and best possible sensitivity towards a virtual lesion.

Another limitation of our study is the degree of disease severity of MS patients. While LTD-like plasticity may be retained in mild-to-moderately affected MS patients, it cannot be ruled out that LTD-like plasticity may be reduced in patients with more severe CNS injury.

## Conclusion

In conclusion, taking into consideration that neither LTP- [9], nor LTD-like (this study) rapid-onset central motor plasticity is impaired in mild-to-moderately affected MS patients, rehabilitation efforts may need to focus on mechanisms supporting the later rather than the early stages of central motor plasticity.

## Competing interests

The authors declare that they have no competing interests regarding this study.

## Authors’ contributions

DZ: study concept and design, acquisition of data, statistical analysis, drafting and revising the manuscript. SYD: acquisition of data, analysis and interpretation of data. DW: acquisition of data, analysis and interpretation of data. PR: study concept and design, analysis and interpretation of data. KVT: analysis and interpretation of data, drafting and revising the manuscript. JC: study concept and design, statistical analysis, drafting and revising the manuscript, study supervision and coordination, obtaining funding. All authors read and approved the final manuscript.

## Pre-publication history

The pre-publication history for this paper can be accessed here:

http://www.biomedcentral.com/1471-2377/12/92/prepub
